# Description of a new species of *Haemogamasus* (Mesostigmata, Laelapidae, Haemogamasinae) from Chubut, Río Negro and Neuquén Provinces, Argentina

**DOI:** 10.3897/zookeys.173.1592

**Published:** 2012-03-02

**Authors:** C. Selby Herrin, Richard D. Sage

**Affiliations:** 1 (CSH) Monte L. Bean Life Science Museum, Brigham Young University, 290 MLBM, P.O. Box 20200, Provo, Utah 84602–0200, U.S.A. (e–mail: sherrin@byu.edu); 2 (RDS) Museum of Vertebrate Zoology, University of California, Berkeley, California 94720, U.S.A.

**Keywords:** ectoparasites, mites, Laelapidae, *Haemogamasus*, Argentina

## Abstract

A new species of *Haemogamasus* mites is described from Chubut, Neuquén and Río Negro Provinces, Argentina. It was collected primarily from rodents of the genus *Abrothrix* (65 of 77 collections): *Abrothrix longipilis* (63), *Abrothrix olivaceus olivaceus* (1) and *Abrothrix olivaceus xanthorhinus* (1). Additional collections were made from *Geoxus valdivianus* (5) and *Loxdomtomys micropus* (5). Possibly incidental or contaminate collections were recorded from *Oligoryzomys longicaudatus* (1) and *Dromiciops gliroides* (1). Most collections came from Río Negro Province (36), with 22 from Neuquén and 19 from Chubut. An identification key is provided to distinguish ♀s of this new species from ♀s of species from the western hemisphere, based on morphological characters.

## Introduction

Mites were collected by the junior author (RDS) during natural history studies of small mammals conducted in the Chubut, Neuquén and Río Negro Provinces, Argentina. Among those collected was a new species belonging to the genus *Haemogamasus* Berlese, 1889. This new species, *Haemogamasus alongipilis*, was collected primarily from rodents of the genus *Abrothrix* (65 of 77 collections): *Abrothrix longipilis* (Waterhouse) (63), *Abrothrix olivaceus olivaceus* (1) and *Abrothrix olivaceus xanthorhinus* (1). Additional collections were made from *Geoxus valdivianus* (5) and *Loxdomtomys micropus* (5). Possibly incidental collections were made from *Oligoryzomys longicaudatus* (1) and *Dromiciops gliroides* (1). The most specimens were taken from Río Negro Province (36), with 22 from Neuquén and 19 from Chubut. Mammal synonymies follow those of Wilson and Reeder (2005).

In his major taxonomic classification treatment of Haemogamasinae, Keegan (1951) separated the subfamily into two closely related genera, *Haemogamasus* and *Euhaemogamasus*. This was based on a major diagnostic character described by Keegan as follows: “The genus *Haemogamasus* is distinguished from *Euhaemogamasus* only in that its species possess accessory sternal setae.” More recent authors have combined the two genera into the single genus *Haemogamasus*. Strandtmann and Wharton (1958) stated: “A fine review of this family [Haemogamasidae] was written by Keegan (1951), a paper from which we have borrowed freely. We have followed Asanuma (1952) and placed *Euhaemogamasus* in synonymy with *Haemogamasus*.” The new species described herein, *Haemogamasus alongipilis*, fits within the designation of the genus *Haemogamasus* as defined by Keegan, as well as the classification followed by Asanuma and other subsequent authors.

In his studies of North American *Haemogamasus liponyssoides*, Radovski (1960) found 4 species with a distinguishing character that differs from all other *Haemogamasus* species. He found that *Haemogamasus liponyssoides*, *Haemogamasus harperi*, *Haemogamasus occidentalis*, and *Haemogamasus keegani* all have slender, untoothed chelae, and concluded that “They appear to constitute a closely related group within the genus, and are referred to here as the *Haemogamasus liponyssoides* complex.”

In a more recent treatment of the *Haemogamasus* species of North America, Williams et al. (1978) presented a new classification for the genus. They placed the North American species into two groups: The *liponyssoides* group and the *reidi* group based the same morphological character as Radovski, plus several additional characters. In their classification the *liponyssoides* group is separated from the *reidi* group by the following characters: Slender, untoothed chelae; anal shield elongate pyriform; and some setae of gnathosoma, sternal shield, anal shield and legs unbarbed. They placed the 4 Radovsky species (*Haemogamasus liponyssoides*, *Haemogamasus harperi*, *Haemogamasus occidentalis*, and *Haemogamasus keegani*), plus a new species, *Haemogamasus ghani*, into the *liponyssoides* group and all other North American species (*Haemogamasus ambulans*, *Haemogamasus pontiger*, *Haemogamasus longitarsus*, *Haemogamasus thomomysi*, *Haemogamasus onychomydis* and *Haemogamasus reidi*) in the *reidi* group. Species of the *reidi* group are differentiated by the following characters: Stout strongly toothed chelae; anal shield broadly pyriform; and some setae on gnathosoma, sternal shield, anal shield and legs barbed.

We have chosen to follow the classification proposed by Williams et al. (1978). The new species described herein, *Haemogamasus alongipilis*, clearly fits within the *liponyssoides* group based on the above described morphological characters. *Haemogamasus alongipilis* is easily distinguished from the previously described *liponyssoides* group by the following characters: Significantly smaller body size (dorsal shield *ca.* 538 µ median long and *ca.*272 µ greatest width); anal shield with no accessory setae; genitoventral shield shorter, not expanded posterior to gv1 setae and with only 1 accessory seta.

The structural morphology and body setae nomenclature followed herein is basically from Keegan (1951), which was subsequently refined by Lindquist and Evans (1965) and Evans and Till (1965, 1966, 1979). The basic leg segmental setae nomenclature and setae formula followed is from Evans (1963), which was subsequently revised by Evans and Till (1966, 1969) and Masan and Fender (2010).

## Materials and methods

Mammals were trapped with Museum Special snap traps (Woodstream Corporation®) baited with oatmeal. Traps were set out in the late afternoon and examined in the morning. Each trapped animals was placed in a plastic bag for later processing for ectoparasites. Ectoparasites were collected by exposing each animal to a stream of carbon dioxide while vigorously brushing over a funnel supported by a ring stand. A 7.5 cm × 15 cm plastic zip–lock bag containing 2 ml of 80% ethanol was affixed with tape to the bottom of the funnel. Ectoparasites (fleas, lice, mites, and staphylinid beetles) fell into the slick–walled funnel and were captured and preserved in the zip–lock bag containing an RDS–field number for the respective mammal host. Sealed bags were subsequently stored for longer periods in a bottle containing 80% ethanol. The funnel was thoroughly cleaned after each animal to preclude cross contamination of ectoparasites from one animal to the next.

Mite specimens were cleared in a solution of KOH and then put through a series of: distilled water, 70%, 85%, 95% & 100% ethyl alcohol, then methyl salicylate and finally xylene. The specimens were then mounted on slides in Canada balsam, and the slides were cured in a warming oven for several weeks in preparation for examination and identification.

Digital images in TIFF format were prepared by photographing with an Olympus BX61 Compound Microscope, Olympus CV12 digital camera using an Olympus Microsuite™ B3SV program. This photograph imaging system was also used to perform all measurements for the detailed descriptions of both ♀s and deutonymphs. All measurements are given in micrmeters (mµ) and all “µ” in the description. In many cases the measurement range is given as “*ca. x*”. We found that there is about a 5–10% variation in measurements of the same structure from specimen to specimen.

The TIFF images were cropped, enhanced, sized appropriately and saved in TIFF as well as JPG format with Adobe Photoshop CS5 Extended version 11.1. Line drawings illustrating diagnostic characters were prepared by printing the TIFF digital images, then tracing them on a light box, scanning the traced images and making necessary adjustment, including setae labeling, with Adobe Photoshop CS5.

The following acronyms for repositories may be associated with material listed under “Type Material” and under “Other Material Examined”: BYU—M. L. Bean Life Science Museum, Brigham Young University, Provo, Utah; MACN—Museo Argentino Bernardino Rivadavia de Ciencias Naturales, Buenos Aires, Argentina; and MCNLP—Museo de Ciencias Naturales de La Plata, La Plata, Argentina.

## Results

### Haemogamasus alongipilis

Herrin & Sage sp. n.

urn:lsid:zoobank.org:act:64F6027D-FC35-4638-AE03-8ED1FD954305 

http://species-id.net/wiki/Haemogamasus_alongipilis

Figs 1 [Fig F2] [Fig F3] [Fig F4] [Fig F5] [Fig F6] [Fig F7] [Fig F8] [Fig F9] [Fig F10] [Fig F11] [Fig F12] [Fig F13] [Fig F14] [Fig F15] [Fig F16] [Fig F17] [Fig F18] 22

#### Type materia.

Holotype, ♀: Argentina: Neuquén Province, Los Lagos Departamento, 4.8 km W, 12.2 km N Villa La Angostura (Parque Nacional Nahuel Huapi), elev. 840 m, (39°02'12.48"S, 70°18'25.68"W), trapline 9, mature Coíhue forest with bamboo, ex. *Abrothrix longipilis*, ♀, 07 XI 2006, R. D. Sage (slide RDS–18117, MACN); paratype, ♀: (same data as holotype, slide RDS–18117, MACN); paratypes, deutonumphs (2): (same data as holotype, slide RDS–18117, MACN); paratypes, ♀s (3) & deutonymphs (3): Argentina: Neuquén Province, Los Lagos Departamento, 3.5 km W, 12.8 km N Villa La Angostura (Parque Nacional Nahuel Huapi), elev. 830 m, trapline 10, mixed Coíhue/Ñire forest with bamboo, *Abrothrix longipilis*, 1 ♂ & 1 ♀, 6 XI 2006, R. D. Sage (slides RDS–18104 & 18106, MCNLP); paratype ♀: Neuquén Province, Aluminé Departamento, 3.8 km W, 0.7 km N Quillén, (Parque Nacional Lanín) elev. 980 m, (39°03'07.26"S, 70°20'42.24"W), trapline 4, Roble Pellín/Ñire forest with bamboo, ex. *Abrothrix longipilis* (Waterhouse), ♀, 13 X 2006, R. D. Sage (slide RDS–18030, BYU); paratypes, ♀s (2) & deutonymphs (4): Neuquén Province, Aluminé Departamento, 3.9 km W, 1.1 km N Quillén (Parque Nacional Lanín), elev. 1010 m, (39°03'07.26"S, 70°20'42.24"W), trapline 3, Roble Pellín forest with bamboo, ex. *Abrothrix longipilis* (Waterhouse), 2 ♂s, 17 X 2006, R. D. Sage (slide RDS–18039 & 18040, BYU); paratype, deutonymph: Argentina, Neuquén Province, Los Lagos Departamento, 4.8 km W, 12.2 km N Villa La Angostura (Parque National Nahuel Huapi), elev. 840 m, (39°02'12.48"S,), trapline 9, mature Coíhue forest with bamboo, ex. *Geoxus valdivianus* Philippi, 1 ♂, 07 XI 2006, R. D. Sage (slide RDS–18112, BYU); paratype, deutonymph (1): Argentina, Neuquén Province, Los Lagos Departamento, 4.8 km W, 12.2 km N Villa La Angostura (Parque National Nahuel Huapi), elev. 840 m, (39°02'12.48"S, 70°18'25.68"W), trapline 9, mature Coíhue forest with bamboo, ex. *Loxdontomys micropus* (Waterhouse), ♂, 07 XI 2006, R. D. Sage (slides RDS–18113, BYU); paratypes, ♀ (1) & deutonymphs (10): Argentina, Neuquén Province, Los Lagos Departamento, 4.8 km W, 12.2 km N Villa La Angostura (Parque National Nahuel Huapi), elev. 840 m, (39°02'12.48"S, 70°18'25.68"W), trapline 9, mature Coíhue forest with bamboo, ex. *Abrothrix longipilis* (Waterhouse), 3 ♂s & 2 ♀s, 07 XI 2006, R. D. Sage (slides RDS–18114, 18116, 18117, 18118 & 18119, BYU); paratype, deutonymph: Argentina, Neuquén Province, Los Lagos Departamento, 4.8 km W, 12.2 km N Villa La Angostura (Parque National Nahuel Huapi), 840 m elev. (39°02'12.48"S, 70°18'25.68"W), trapline 9, mature Coíhue forest with bamboo, ex. *Geoxus valdivianus* Philippi, 1 ♀, 8 XI 2006, R. D. Sage (slide RDS–18121, BYU).

**Table 1.** Other Material Examined (ordered by locality).

1	Trapline 3, Roble Pellín forest with bamboo, 3.9 km W, 1.1 km N Quillén (Parque Nacional Lanín), elev. 1010 m, Aluminé Dpto., Neuquén Prov., Argentina, Richard D. Sage	17 X 2006: RDS–18040 *Abrothrix longipilis* (♂) –1 deutonymph.15 X 2007: RDS–18569, 18572 & 18573 *Abrothrix longipilis* (2 ♀s & 2 ♂s) – 4 deutonymphs.
2	Trapline 4, Roble Pellín/Ñire forest with bamboo, 3.8 km W, 0.7 km N Quillén (Parque Nacional Lanín), elev. 980 m, Aluminé Dpto., Neuquén Prov., Argentina, Richard D. Sage	14 X 2007: RDS–18565 *Abrothrix longipilis* (♀) – 1 deutonymph.16 X 2007: RDS–18565 *Geoxus valdivianus* (♀) – 1 deutonymph.
3	Trapline 9, Coíhue forest with bamboo, 4.8 km W, 12.2 km N Villa La Angostura (Parque Nacional Nahuel Huapi), elev. 840 m, Los Lagos Dpto., Neuquén Prov., Argentina, Richard D. Sage	7 XI 2006: RDS–18112 *Geoxus valdivianus* (♂) – 1 deutonymph; RDS–18113 *Loxdontomys micropus* (♂) – 1 deutonymph.8 XI 2006: RDS–18121 *Geoxus valdivianus* (♀) – 1 deutonymph.
4	Trapline 10, mixed Coíhue/Ñire forest with bamboo, 3.5 km W, 12.8 km N Villa La Angostura (Parque Nacional Nahuel Huapi), elev. 830 m, Los Lagos Dpto., Neuquén Prov., Argentina, Richard D. Sage	6 XI 2006: RDS–18102 *Abrothrix longipilis* (♂) – 1 deutonymph; RDS–18103 *Abrothrix olivaceus olivaceus* (♂) – 1 deutonymph.
5	Trapline 6, in green bamboo/Coíhue/Ñire forest on Península Llao Llao, 0.4 km N of Parkguard station between Lagos Perito Moreno and Escondido (Parque Municipal Llao Llao), elev. 831 m, Bariloche Dpto., Río Negro Prov., Argentina, Richard D. Sage	26 XI 2006: RDS–18144, 18145, 18148 & 18153 *Abrothrix longipilis* (3 ♂s & 1 ♀) – 4 deutonymphs.27 V 2007: RDS–18433 & 18437 *Abrothrix longipilis* (1 ♂ & 1 ♀) – 2 ♀s, 1 deutonymph.29 V 2007: RDS–18471 & 18475 *Abrothrix longipilis* (2 ♀s) – 4 deutonymphs.23 X 2007: RDS–18581 *Loxdontomys micropus* (♀) – 1 deutonymph; RDS–18585, 18586, 18588 & 18598, *Abrothrix longipilis* (2 ♀s & 2 ♂s) – 5 deutonymphs; RDS–18593 *Oligoryzomys longicaudatus* (♀) – 2 deutonymphs.
6	Trapline 7, in green bamboo/Coíhue/Ñire forest on Peninsula Llao Llao, 0.3 km W of Parkguard station between Lagos Perito Moreno and Escondido (Parque Municipal Llao Llao), elev. 800 m, Bariloche Dpto., Río Negro Prov., Argentina, Richard D. Sage	6 XI 2006: RDS–18127, 18128 *Geoxus valdivianus* (2 ♀s) – 2 ♀s & 1 deutonymph.25 XI 2006: RDS–18130, 18131, 18132 & 18136 *Abrothrix longipilis* (1 ♂ & 3 ♀s) – 4 deutonymphs.28 V 2007: RDS–18442 *Loxodontomys micropus* (♂) – 3 deutonymphs; RDS–18449, 18453, 18455, 18456 & 18461 *Abrothrix longipilis* (3 ♂s & 2 ♀s) – 9 ♀s & 7 deutonymphs.1 XI 2007: RDS–18604 *Dromiciops gliroides* (♀) – 1 ♀; RDS–18608, 18609, 18610, 18612, 18615, 18616, 18620, 18621 & 18624 *Abrothrix longipilis* (5 ♂s & 3 ♀s) – 11 ♀s & 13 deutonymphs.
7	Trapline 1, mixed Ñire/Ciprés forest with bamboo, 5.5 km S, 2.8 km E Villa Futalaufquen (Parque Nacional Los Alerces), elev. 547 m, Futaleufú Dpto., Chubut Prov., Argentina Richard D. Sage	26 X 2006: RDS–18071, 18072, 18078, 18079, 18082, 18083 & 18084 *Abrothrix longipilis* (4 ♂s, 3 ♀s) – 9 deutonymphs.26–27 X 2006: RDS–18069 & 18078 *Loxdontomys micropus* (2 ♂s) – 3 deutonymphs.13 XI 2007: RDS–18658 *Abrothrix longipilis* (♂) – 1 deutonymph.
8	Trapline 2, Ñire forest with bamboo, 1.5 km S, 0.5 km E Villa Futalaufquen (Parque Nacional Los Alerces), elev. 590 m, Futaleufú Dpto., Chubut Prov., Argentina Richard D. Sage	25 X 2006: RDS–18059 *Abrothrix olivaceus xanthorhinus* (♂) –1 deutonymph; RDS–18060 *Abrothrix longipilis* (♂) – 1 deutonymph; RDS–18066 *Abrothrix longipilis* (♀) – 2 deutonymphs.10 XI 2007: RDS–18628, 18630, 18633, 18635, 18638, 18640 & 18645 *Abrothrix longipilis* (6 ♂s & 2 ♀s) – 2 ♀s & 5 deutonymphs.

#### Diagnosis.

♀s of *Haemogamasus alongipilis* possess a number of distinguishing characters: (1) Small body size (dorsal shield *ca.* 538 µ median long and *ca.*272 µ greatest width); (2) chelicera chelae (see Fig. 4) slender with untoothed (edentate) chelae; (3) sternal shield (see Fig. 4) approximately square in shape (length/width ratio 0.98), extending posteriorly to level of mid coxae III; (4) sterna shield with 6 pair plus 1 (13) accessory setae in addition to the usual 3 pair of setae; (5) no accessory setae on anterior margin of sterna shield (only st-1 setae on anterior margin); (6) gnathosomal, sternal shield, anal shield and leg setae smooth, none barbed; (7) posterior margin of sterna shield only slightly concave; (8) genitoventral shield (see Fig. 3) not unusually expanded posterior to coxae IV; (9) genitoventral shield with only 5 setae: The usual gv1 and gv2 pairs, plus 1 accessory seta centered at level posterior to gv2; (10) anal shield (see Fig. 2) with no accessory setae; and (10) the usual pair of paranal setae positioned well posterior to the anal orifice.

**Figures 1–4.**
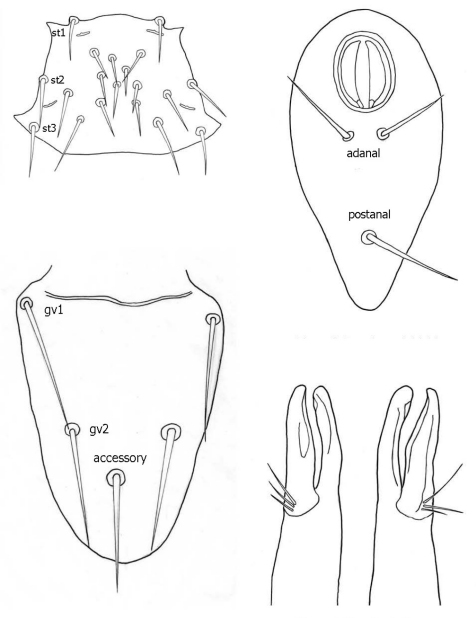
**1** Female sternal shield **2** Female anal shield **3** Female genitoventral shield **4** Female chelicerae.

#### Differential diagnosis.

♀s of *Haemogamasus alongipilis* are separable from all Western Hemisphere *Haemogamasus* species by the following combination of characters: (1) Significantly smaller body size (dorsal shield *ca.* 538 µ median long and *ca.*272 µ greatest width); (2) sternal shield (see Fig. 1) approximately square in shape (length/width ratio 0.98); (3) No accessory setae on the anal shield (see Fig. 2); (4) paranal setae positioned at a level posterior to the anal orifice; and (5) genitoventral shield (see Fig. 3) shorter, not expanded posterior to gv1 or gv2 setae and with only 1 accessory seta centered at level posterior to gv2 seta. *Haemogamasus alongipilis* and *Haemogamasus harperi* share a single diagnostic character: Both have sternal shield accessory setae which distinguishes them from all other western hemisphere species.

Thus far, no ♂s of *Haemogamasus alongipilis* are known from the 77 collections in Argentina, probably because males are rarely collected; however many deutonymphs were collected and are described herein. It is interest to note that of the 77 collections, there were 99 deutonymphs collected whereas there were only 37 ♀s collected. This ratio of deutonumphs to females (3:1) is likely because the deutonymph stage is the migrating stage. This species is named after the predominant rodent host species, *Abrothrix longipilis*, from which 65 of the 77 collections were made.

The diagnostic key below for ♀s of the Western Hemisphere is adapted from Williams et al. (1978) to show the relationship of *Haemogamasus alongipilis* with other species. It shows *Haemogamasus alongipilis* separable from other *Haemogamasus* species based on significant morphological characters (Figs 1–4) with notes on geographical records.

#### Diagnostic key to female of the western hemisphere.

**Table:** 

1	Chlicerae slender with un-toothed chelae (edentate); anal shield elongate pyriform; no barbed setae on gnathosoma, sternal shield, anal shield and legs (*liponyssoides* group)	2
–	Chlicerae stout with strongly toothed chelae (dentate); anal shield broadly pyriform; some setae on gnathosoma, sternal shield, anal shield and legs barbed (*reidi* group)	8
2	Sternal shield with accessory setae	3
–	Sternal shield without accessory setae	4
3.	Anal shield without accessory setae; genitoventral shield with only 1 accessory seta; dorsal shield length/width *ca.* 538 µ /*ca.*272 µ [Patagonia, Argentina]	*Haemogamasus alongipilis* sp. n.
–	Anal shield with accessory setae; genitoventral shield with 11-18 accessory setae; dorsal shield length/width *ca.* 966 µ /*ca.5*70 µ [southeastern USA]	*Haemogamasus harperi* Keegan
4	Peritreme short (*ca.* 100 µ); genitoventral shield with 11-15 accessory setae on posterior one half of shield; tarsi abruptly narrowed distally [western USA]	*Haemogamasus keegani* (Jameson)
–	Peritreme longer (*ca.* 200 µ); genitoventral shield with more than 22 accessory setae on posterior one half of shield; tarsi not abruptly narrowed distally	5
5	Tectum with long distal and long lateral fimbriae; deutosternal groove narrow with one to eight teeth in anterior rows	6
–	Tectum with long distal and short lateral fimbriae; deutosternal groove broad with eight or more teeth in anterior rows	7
6	Sternal shield with posterior margin invaginated to level about midway between sternal setae II and III [eastern USA, southern Canada & Holarctic]	*Haemogamasus liponyssoides liponyssoides* Ewing
–	Sternal shield with posterior margin invaginated only to level of sternal setae III [California]	*Haemogamasus liponyssoides hesperus* Radovsky
7	Sternal shield with posterior margin nearly straight; genitoventral shield with 30 or more accessory setae; *pilus dentilis* and dorsal seta present [northwestern USA & southwestern Canada]	*Haemogamasus occidentalis* Keegan
–	Sternal shield with posterior margin invaginated nearly to level of sternal setae III; genitoventral shield with less than 20 accessory setae; *pilus dentilis* and dorsal seta absent [Alaska, USA]	*Haemogamasus ghanii* Williams et al.
8	Sternal shield with accessory setae; hypostomal setae I unbarbed; *pilus dentilis* setiform [Canada & USA and worldwide	*Haemogamasus ambulans* (Thorell)
–	Sternal shield without accessory setae; hypostomal setae I barbed or unbarbed; *pilus dentilis* variable	9
9	Sternal shield with posterior margin invaginated to between sternal setae I and II; hypostomal setae I unbarbed; setation of dorsal and genitoventral shields sparse; legs II-IV with relatively stout ventral setae [USA & Canada and worldwide]	*Haemogamasus pontiger* (Berlese)
–	Sternal shield with posterior margin never invaginated to or above sternal setae II; hypostomal setae I barbed; setation of dorsal and genitoventral shields dense; legs without stout ventral setae	10
10	Genitoventral shield very broad, flask-shaped, width about one-half length	11
–	Genitoventral shield shape variable, never extremely broad, width usually about one-third length	12
11	Genitoventral shield with accessory setae extending anteriorly to flap of shield; *pilus dentilis* very long, expanded laterally [eastern USA & Canada]	*Haemogamasus longitartus* (Banks)
–	Genitoventral shield with accessory setae only on posterior two-thirds of shield, none past genital setae; *pilus dentilis* long and basally swollen [California & Utah, USA]	*Haemogamasus thomomysi* Williams et al.
12	*Pilus* dentilis short, setiform; large corniculi (*ca.* 65 long); chelicerae with a conspicuous brush border at base; leg setae thickly barbed; most dorsal setae barbed [western USA]	*Haemogamasus onychomydis* (Ewing)
–	*Pilus* dentilis long, curved distally; smaller corniculi (*ca.* 30 long); no brush border at base of chelicerae; leg setae sparsely barbed; only posterior and marginal dorsal setae barbed [USA & Canada]	*Haemogamasus reidi* Ewing

#### Description of the female.

Figures 5-13

**Figure 5. F2:**
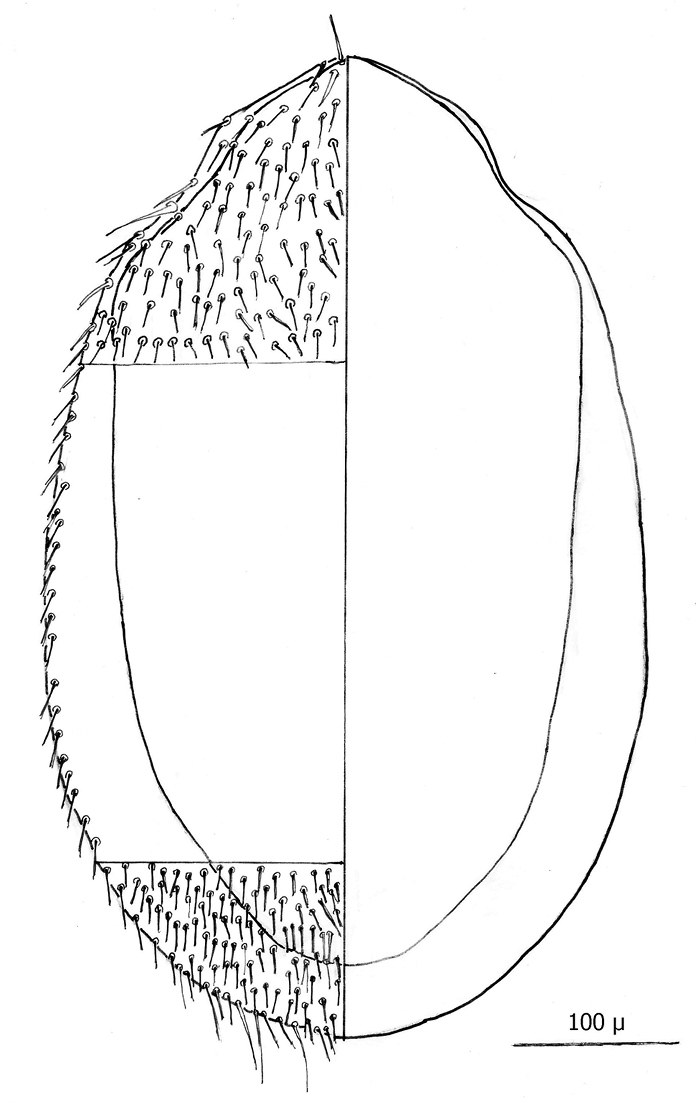
Line drawing of ♀ dorsum.

**Figure 6. F3:**
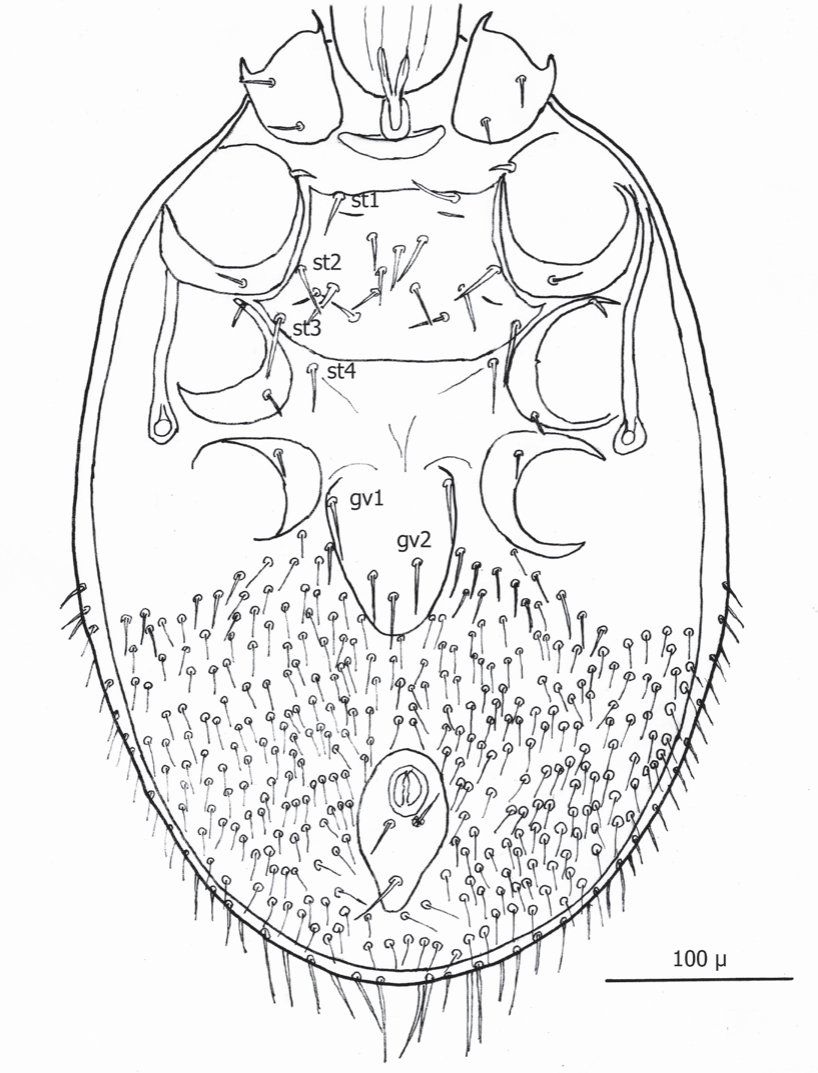
Line drawing of ♀ venter.

The following two line drawings show the general shape of the body as well as the most distinctive morphological characters. The dorsal view (Fig. 5) shows the outline of the dorsal shield with an anterior-lateral and a posterior-lateral cutout showing the relative size and number of setae. The ventral view (Fig. 6) shows the outline of the ventral shields with the relative size and number of setae associated with each.

**Figure 7. F4:**
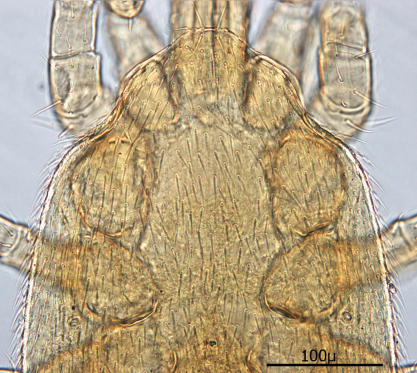
Anterior dorsum.

**Figure 8. F5:**
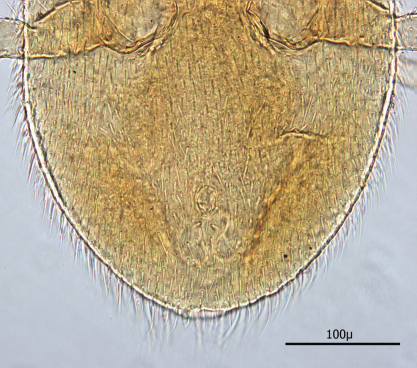
Posterior dorsum.

*Dorsum* (Figs 7–8): Body elliptical with dorsal shield length/width ratio *ca.* 0.98; dorsal shield median length *ca.* 538 µ (515 –576 µ) and greatest width *ca.* 272 µ (250 –294 µ); anterior margin acutely rounded; lateral margins at level of anterior coxa I slightly concave; lateral margins at level between coxa I and coxa II moderately concave forming a rounded shoulder at level of coxa I; median lateral margins almost straight to slightly rounded; posterior lateral margins more strongly rounded; and posterior margin broadly rounded. Dorsal shield bearing numerous setae, far too many to count (see Figs 5–8). Marginal setae setaceous (*ca.* 24–26 µ long); medial setae setaceous (*ca.* 16–26 µ long); posterior terminal pair of setae (Z5) long and slender (*ca.* 54 µ); and J5 setae (just anterior to Z5) setaceous and longer (*ca.* 60 µ).

**Figure 9. F6:**
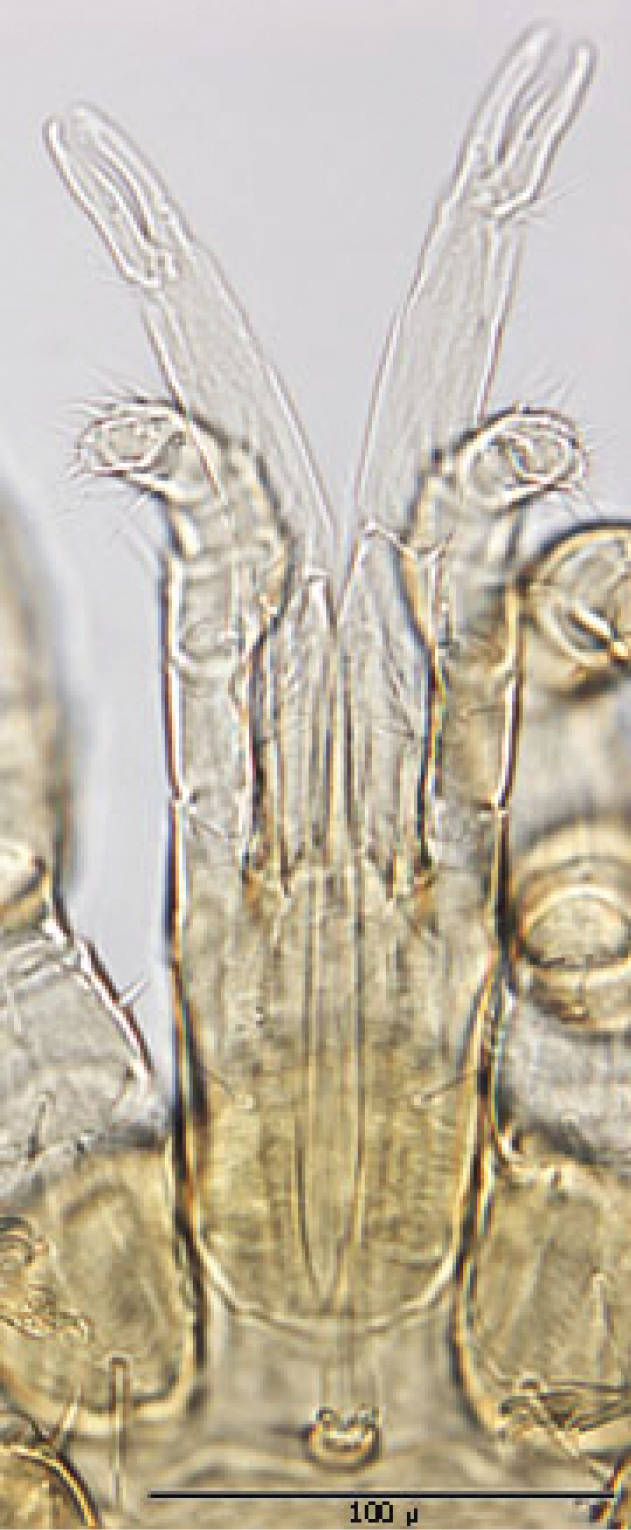
Ventral gnathosoma.

*Gnathosoma* (Figs 9): Greatest width at level of gnathosomal setae *ca.* 81 µ, and length from base to palpal trochanter *ca.* 114 µ. Gnathosomal setae setaceous and small, length *ca.* 19 µ; lateral hypostomal setae slightly longer (*ca.* 22 µ); medial hypostomal length *ca.* 39 µ; and distal hypostomal setae length *ca.* 18 µ. Distance between gnathosomal setae *ca.* 36 µ and distance from medial hypostomal setae to gnathosomal setae *ca.* 58 µ. Chelicera long and slender; moveable digit of chelae edentate; chelae length *ca.* 40 µ, with 2^nd^ cheliceral segment *ca.* 111 µ long. Corniculi small (*ca.* 12-13 µ /3-5 µ) and somewhat horn shaped. Tectum of moderate length with 3 terminal fimbriae, middle much elongated. Deutosternal groove parallel sided with 8-10 transverse rows of tiny denticles. Pedipalps chaetotaxy 2–3–5– + terminal 10-14 tarsal setae; segment length/width as follows: Trochanter 41/20 µ, femur 28/27 µ, tibia 10/22 µ and tarsus 16/22 µ.

**Figure 10. F7:**
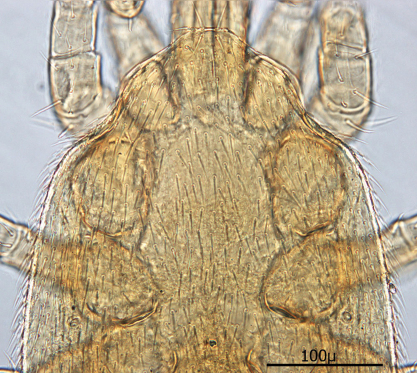
Sternal shield.

**Figure 11. F8:**
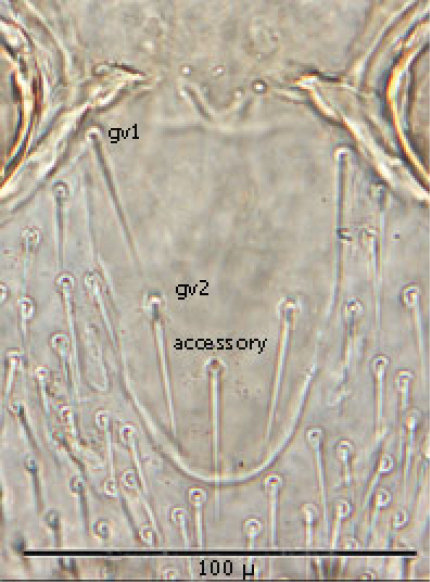
Genitoventral shield.

**Figure 12. F9:**
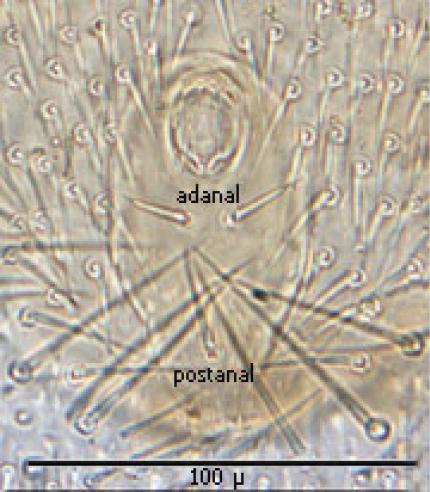
Anal shield.

*Venter* (Figs 10–12): Tritosternum well developed with long, slender laciniae (*ca.* 108 µ) with base *ca.* 29 µ long and 17 µ wide. Presternal area reticulate, with no apparent minute spines.

Sternal shield (Fig. 10) generally square in shape extending posteriorly to level of mid coxae III; length/width ratio 0.98; median length *ca.* 99 µ and width between coxae II *ca.* 101 µ. Anterior margin of sternal shield convex between st1 setae. Posterior margin slightly concave medially between st3 setae. Sternal shield bearing the usual 3 pairs of setae (st1, st2 and st3) plus 7 pairs of accessory setae and 1 single seta between and posterior to level of first pair of accessory setae. The usual 3 pairs of sternal setae are setaceous and subequal in length (*ca.* 36–42 µ). The accessory setae vary in length with anterior medial setae distinctly shorter than the posterior lateral setae.

Setae st1 on the anterior margin with concavities on both sides of each seta. Two pairs of distinct slit–like pores/ trichopores (lyriform in shape) associated with setae st1 and st2; the pair posterior to st1 generally parallel to anterior margin; the pair between st2 and st3 oriented at nearly 45 degree angle with medial end tipped up; third pair off shield on soft integument adjacent to st4.

Distance between st1 setae *ca.* 65 µ; distance between st2 setae *ca.* 116 µ; and distance between st3 setae *ca.* 129–235 µ. Distance between st1 and st2 setae *ca.* 46 µ and distance between st2 and st3 setae *ca.* 38 µ. Metasternal setae (st4) *ca.* 38 µ long; situated on small, oval metasternal shields at level of mid coxae III and just posterior to posterior–lateral margins of sterna shield.

Genitoventral shield (Fig. 11) tongue–shaped with 5 setae (2 pairs plus 1 single seta posterior to level of 2^nd^ pair); gv1 setae positioned laterally adjacent to coxae IV; gv2 setae positioned a little more than half way between the gv2 setae and the posterior end of the genitoventral shield and not near margins; all 5 setae subequal in length (*ca.* 42–43 µ).

Distance between gv1 setae *ca.* 58 µ and distance between gv2 setae *ca.* 33 µ. Lateral margins of genitorventral shield gently converging with posterior end broadly rounded. Genitoventral shield length/width ratio 0.65; least width of the shield between coxae IV 70 µ; greatest width posterior to genitor ventral setae *ca.* 70 µ; greatest length (level between coxae IV to posterior end) *ca.* 100 µ; length from gv1 setae to posterior end *ca.* 82 µ. Parapodal shields posterior to coxae IV on unarmed venter oval to oblong (ca. 32 µ long and 17 µ wide). Unarmed venter thickly beset with setae; more medial setae short (16–23 µ long) and more lateral setae 60–66 µ long.

Anal shield (Fig. 12) not in juxtaposition with genitoventral shield; distance between genitoventral and anal shield *ca.* 86 µ. Anal shield length/width ratio 1.93; anterior margin strongly rounded with lateral margins gently rounded; length from anterior margin to postanal seta *ca.* 98 µ; greatest width at level of anal orifice *ca.* 50 µ; adanal setae situated at level posterior to anal orifice; distance between adanal setae *ca.* 13 µ; adanal setae setaceous *ca.* 22 µ long; distance from anterior margin to adanal setae *ca.* 45 µ; distance from adanal setae to postanal setae *ca.* 34 µ; postanal setae long setaceous *ca.* 33 µ long.

**Figure 13. F10:**
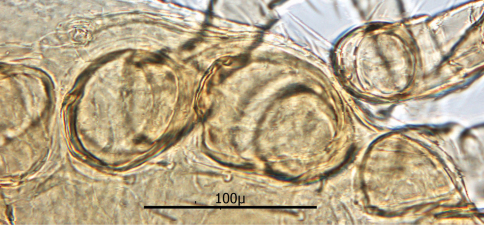
Peritreme.

Shorter setae of unarmed venter length 16–23 µ; longer setae length 60–66 µ. Stigma of peritreme (Fig. 13) located lateral to coxae III and IV with only a short posterior peritremala extension; anterior extension of peritreme narrowing somewhat and reaching anteriorly to level slightly anterior to coxae II; total length of peritreme *ca.* 235 µ.

*Legs* The length and width of leg segments is given in Table 2 below. All measurements were made ventrally. Length measurements were made mid-ventrally and widths were made at the widest point. The measurements are approximate because there is about a 5–10% variation in measurements from specimen to specimen.

The leg segment chaetotaxy for females is shown in table 3 below. The chaetotaxy formula for each leg segment is as follows: al — d/v — pl (anterior laterals — dorsals/ventrals — posterior laterals). The total for each leg segment is in parenthesis bolded.

Leg I: Coxae I greatest width *ca.* 54 µ and mid–ventral length *ca.* 55 µ; distal setae setaceous and *ca.* 25 µ long; proximal setae setaceous and *ca.* 31 µ long. Proximal ventral seta of trochanter I slender setaceous *ca.* 34 µ long, and distal seta *ca.* 26 µ long. Distal dorsal setae of femur I setaceous but with one short (*ca.* 18 µ) and the other almost twice as long (*ca.* 30 µ). Proximal–most dorsal seta of genu I with one short (*ca.* 12 µ) and other longer (*ca.* 28 µ). Tarsus I with all setae setaceous.

Leg II: Coxae II greatest width *ca.* 91 µ and mid–ventral length *ca.* 27 µ; anterior seta setaceous and short (*ca.* 24 µ); posterior seta setaceous and much longer (*ca.* 39 µ). Ventral setae of trochanter setaceous and proximal seta much longer (*ca.* 31 µ) than distal (*ca.* 15 µ). Two distal dorsal setae of femur II slender setaceous and much longer (*ca.* 23–24 µ) than any others. Proximal dorsal setae of genu II setaceous and not unusually long. Tarsus II with all pre–apical seta setaceous.

Leg III: Coxae III greatest width *ca.* 79 µ and mid–ventral length *ca.* 25 µ. Anterior seta setaceous and slightly longer than posterior (*ca.* 25 µ); posterior seta setaceous and shorter (*ca.* 24 µ). Anterior distal setae of trochanter setaceous and *ca.* 27 µ long; posterior seta of trochanter slender setaceous (*ca.* 18 µ long). Setae of femur III all setaceous and of approximate equal length (18–20 µ). All setae of genu III setaceous and not unusually long (16–24 µ). Tarsus III with two pre–apical seta large and more spine–like and others setaceous.

Leg IV: Coxae IV greatest width *ca.* 65 µ and mid–ventral length *ca.* 29 µ; single seta setaceous and short *ca.* 17 µ long. Two anterior distal setae of trochanter setaceous (21–28 µ long); posterior proximal seta setaceous and longer (*ca.* 31 µ); posterior distal seta smaller (*ca.* 22 µ long). Distal setae of femur IV stout setaceous (*ca.* 21 µ long); posterior distal setae setaceous and longer (*ca.* 32 µ). Anterior distal setae of genu IV longer setaceous (*ca.* 24 µ), with all others setaceous and smaller (17–20 µ). Tarsus IV with pre–apical seta stout setaceous and others more slender setaceous.

#### Description of the deutonymph

Figures 14–22

*Diagnostic characters for deutonymphs*:

(1) Body elliptical and significantly larger than ♀s: length *ca.* 707 µ, and width at level of coxae III *ca.* 430 µ.

(2) Dorsal shield divided at level between coxae III and IV, with both dorsal shields thickly beset with setae.

(3) Ventral shield (no separate sternal and genitoventral shields) elongate, extending from level of coxae I to posterior 1/3 of coxae IV and tapering strongly between coxae IV to a blunt point posteriorly.

(4) Ventral shield with 4 pairs of setae, which are the three pairs of usual sternal setae and the single pair of metasternal setae.

(5) Two pairs of ventral setae on soft integument between posterior extension of ventral shield and coxae IV; second pair posterior to ventral shield and adjacent to posterior of coxae IV.

(6) Anal shield somewhat diamond shaped with length and width about equal; adanal setae short not extending to base of postanal seta, which is very long (about 3½ times as long as paranal setae).

**Figure 14. F11:**
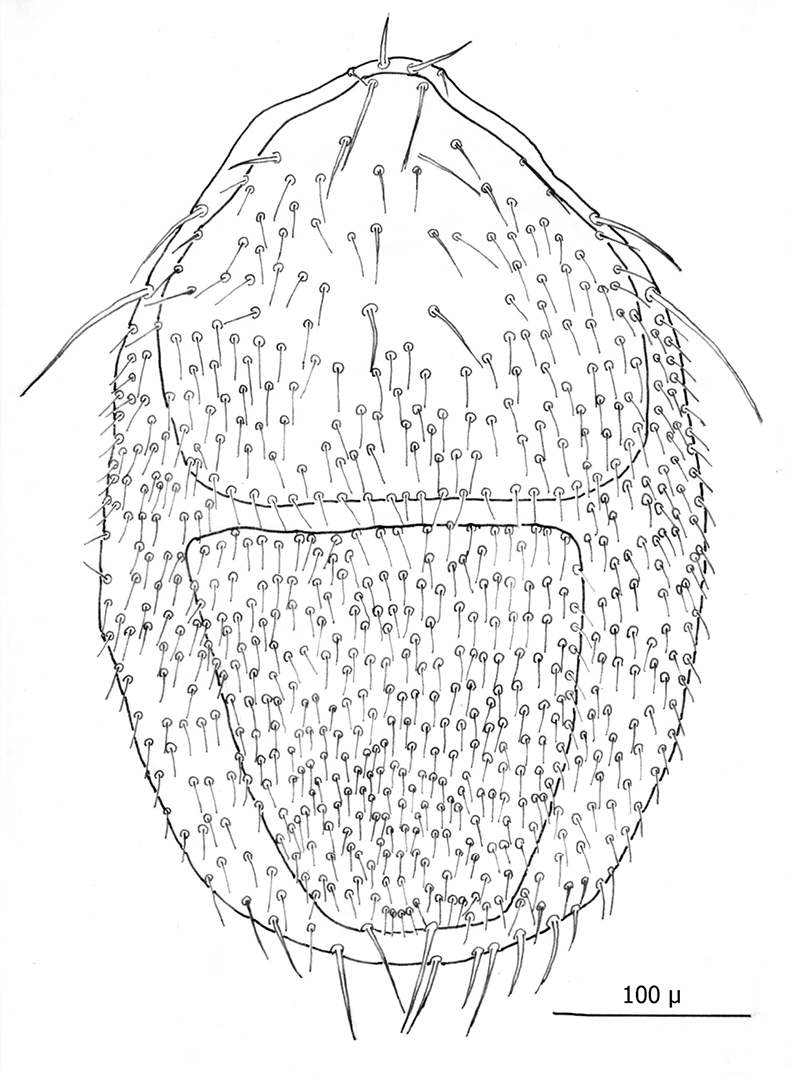
Line drawing of dny dorsum.

**Figure 15. F12:**
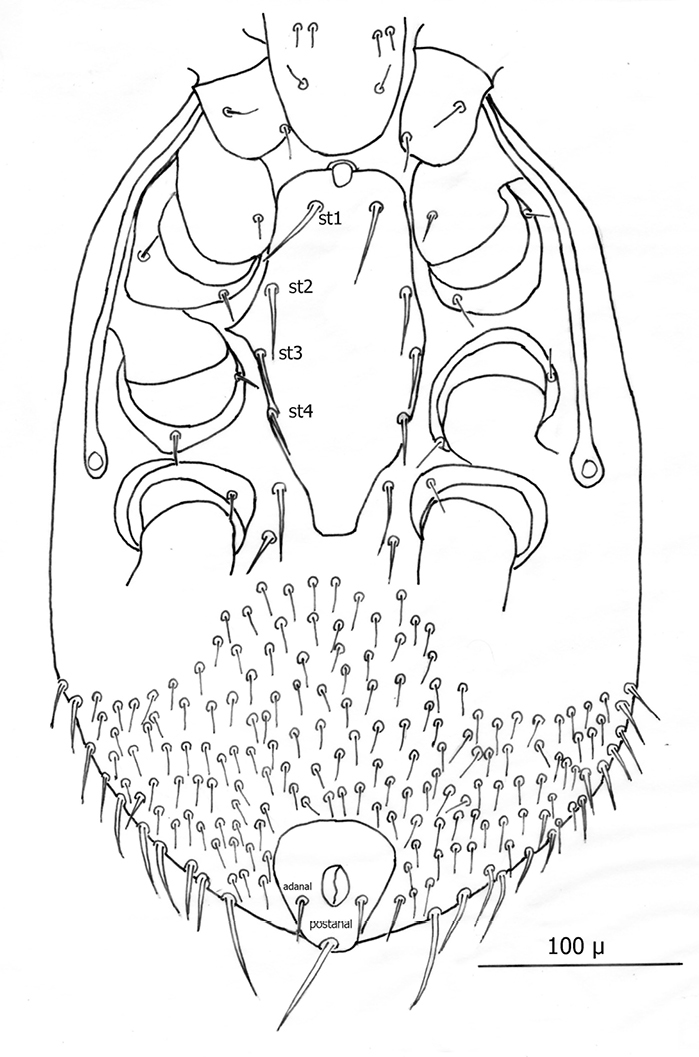
Line drawing of dny venter.

The following two line drawings show the general shape of the body as well as the most distinctive morphological characters. The dorsal view (Fig. 14) shows the outline of the dorsal shields showing the relative size and number of setae. The ventral view (Fig. 15) shows the outline of the ventral shields with the relative size and number of setae associated with each.

**Figure 16. F13:**
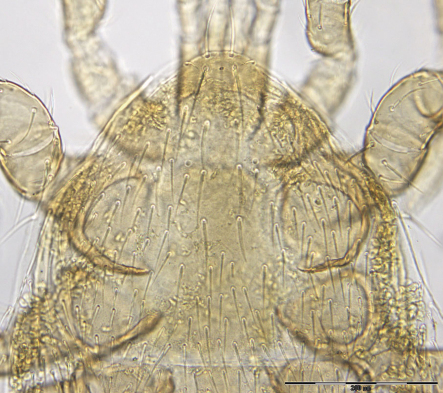
Anterior dorsum.

**Figure 17. F14:**
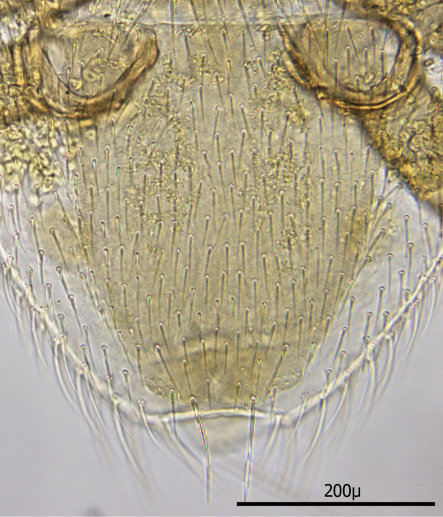
Posterior dorsum.

*Dorsum* (Figs 16–17): Body elliptical with two dorsal shields divided at level between coxae III and IV. Anterior and posterior dorsal shields combined length/width ratio *ca.* 1.75. Anterior dorsal shield length *ca.* 390 µ (371–442 µ) and greatest width *ca.* 447 µ (389–500 µ). Posterior dorsal shield length *ca.* 364 µ (323–402 µ) and greatest width *ca.* 341 µ (328–359 µ). Anterior tip of anterior dorsal shield pointed with only one “shoulder” at level of r4 setae. Anterior–lateral margins somewhat concave between anterior tip and “shoulder”; lateral margins posterior to “shoulder” broadly rounded. All dorsal setae setaceous and of varying lengths: Anterior marginal setae (r2, r3 & r4) shorter (35–43 µ long) than central setae; anterior central setae (i2, i3, i4, s1& s2) 73–91 µ long, and posterior central setae (j2, j3 & j4) shorter (38–42 µ long); Posterior marginal setae somewhat longer than anterior marginal (48–51 µ long); posterior terminal pair of setae (Z5) long and slender (81–82 µ long), and J5 setae (just anterior to Z5) short about same as j2–j4 (34–35 µ long).

**Figure 18. F15:**
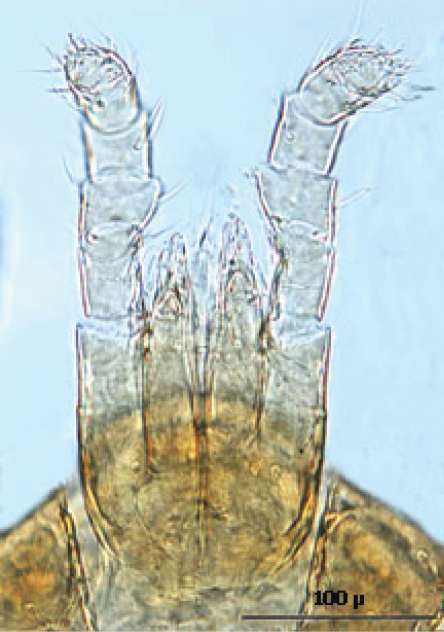
Gnathosoma.

**Figure 19. F16:**
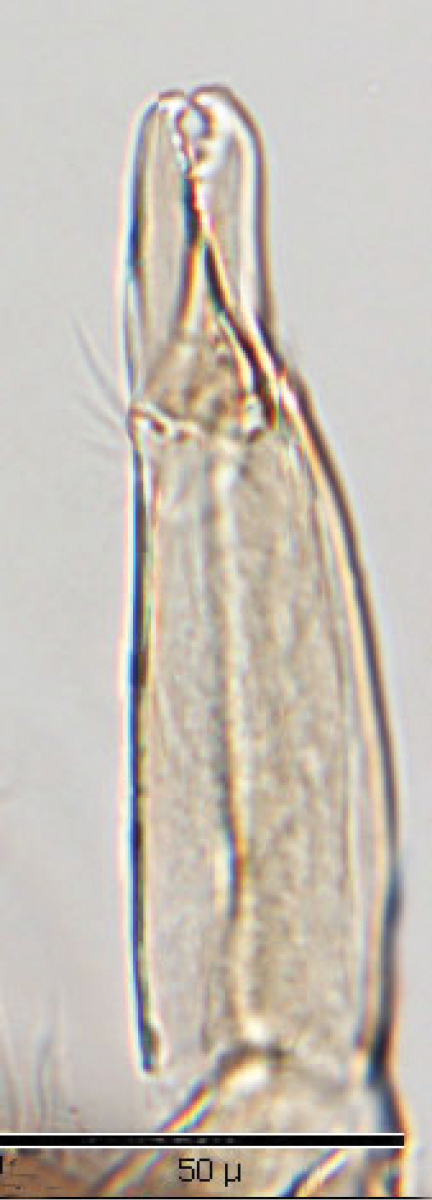
Chelicerae.

*Gnathosoma* (Figs 18—19): Greatest width at level of gnathosomal setae *ca.* 124 µ, and length from base to palpal trochanter *ca.* 119 µ. Gnathosomal setae setaceous *ca.* 56 µ long. Hypostomal setae all setaceous; lateral hyposomal setae *ca.* 24 µ long; medial hyposomal 48–58 µ long; and distal hyposomal setae 20–23 µ long. Distance between gnathosomal setae *ca.* 72 µ, and distance from medial hypostomal setae to gnathosomal setae *ca.* 63 µ. Moveable digit of chelae minutely toothed; chelae *ca.* 37 µ long, with 2^nd^ cheliceral segment *ca.* 116 µ long. Corniculi distinctly horn shaped (*ca.* 22/5 µ). Tectum of moderate length with 3 terminal fimbriae, middle much elongated. Deutosternal groove parallel sided with 14-16 transverse rows of possibly minute denticles. Pedipalps chaetotaxy 2–5–6–9 + terminal 12-14 tarsal setae; segments length/width as follows: Trochanter 29/32 µ, femur 43/39 µ, genu 23/37 µ, tibia 17/30 µ and tarsus 30/30 µ.

**Figure 20. F17:**
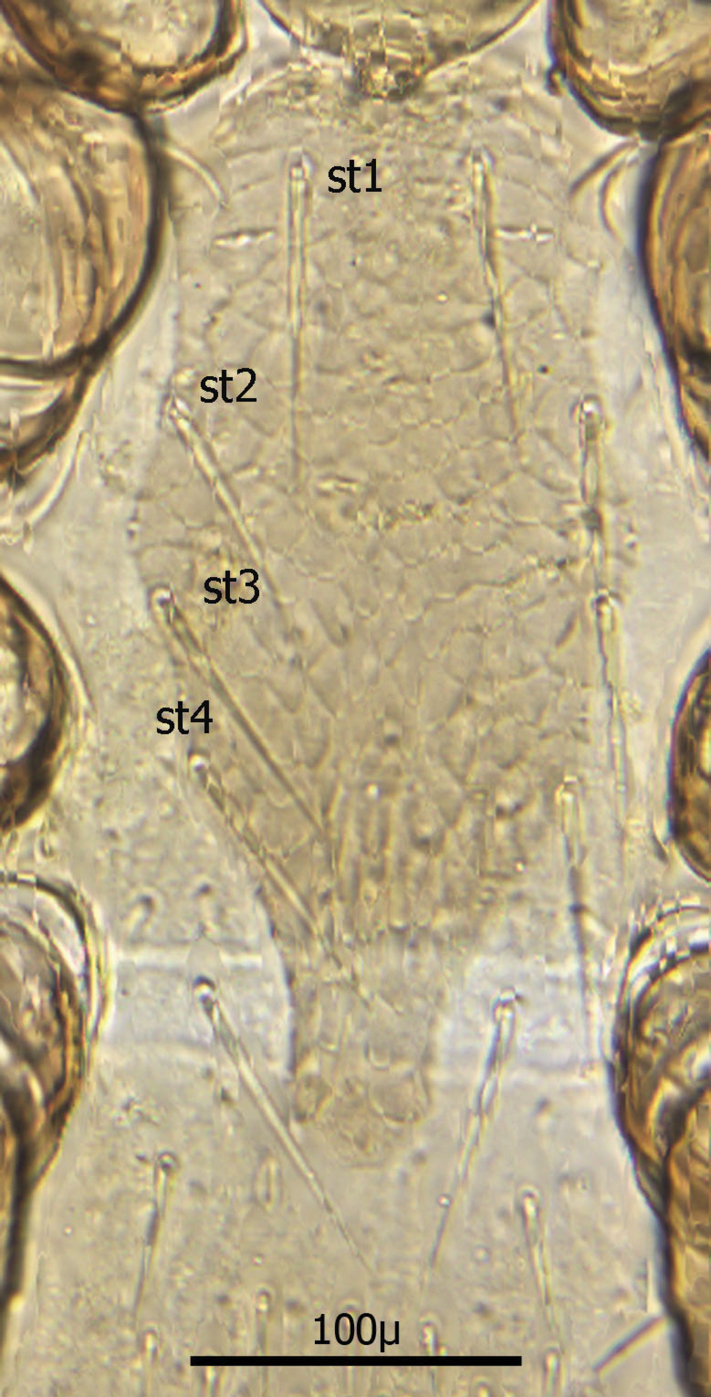
Ventral shield.

**Figure 21. F18:**
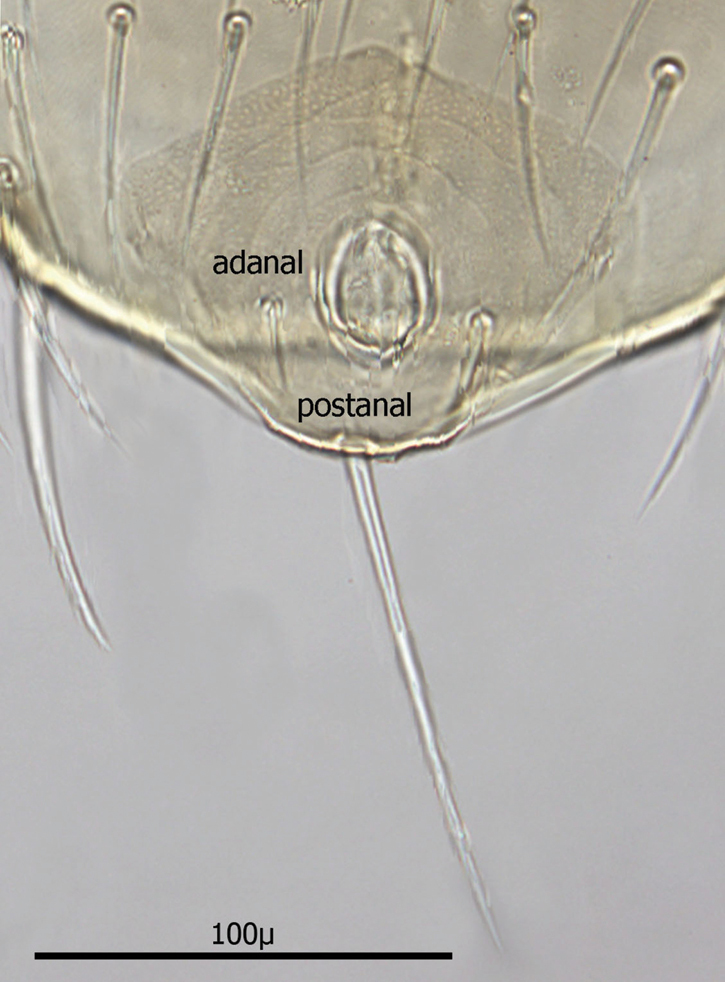
Anal shield.

**Figure 22.**
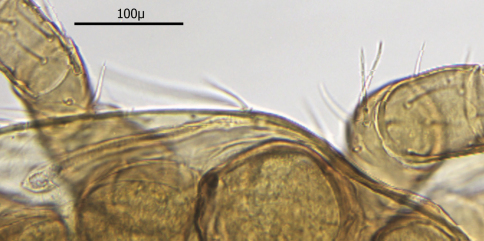
Peritreme.

*Venter* (Figs 20–22): Tritosternum well developed with long, slender laciniae (*ca.* 129 µ) with base *ca.* 11 µ long and 18 µ wide. Deutonymphs do not have separate sternal and genitoventral shields, but rather a single ventral shield that is elongated and extending from level between coxae I and II to level midway between coxae IV. This ventral shield (Fig. 20) has 4 pairs of setae, which represent the three pairs of usual sternal setae and the single pair of metasternal setae of. Ventral shield median length *ca.* 306 µ; width between coxae II *ca.* 147 µ; width between coxae III *ca.* 123 µ; width between coxae IV *ca.* 98 µ. Anterior margin of ventral shield irregular with concavity at level of tritosternum base; posterior end of ventral shield narrowly rounded posterior to a pair of ventral setae that flank the shield on the soft integument. two pair of ventral setae are posterior to ventral shield on the soft integument and adjacent to posterior 1/3 of coxae IV. All ventral shield setae setaceous and rather long: Gv1 setae 96–100 µ; gv2 setae *ca.* 99 µ; gv3 setae *ca.* 100 µ; and gv4 setae 93–94 µ; setae adjacent and posterior to ventral shield ca. 93–94µ long. One pair of slit–like pores/ trichopores (lyriform in shape) posterior to setae gv1; pores not evident adjacent to other ventral shield setae. Distance between gv1 setae *ca.* 51 µ; distance between gv2 setae *ca.* 122 µ; distance between gv3 setae *ca.* 113 µ; and distance between gv4 setae *ca.* 112 µ. Distance between gv1 and gv2 setae *ca.* 78 µ; distance between gv2 and gv3 setae *ca.* 57 µ; and distance between gv3 and gv4 setae *ca.* 50 µ.

Anal shield (Fig. 21) not in juxtaposition with ventral shield; distance between ventral shield and anal shield *ca.* 317 µ. Anal shield somewhat diamond shaped and slightly wider than long with length/width ca. 0.81/0.86 µ; anterior margin acutely rounded and lateral margins more broadly rounded; length from anterior margin to postanal seta 99–100 µ; greatest width at level of anal orifice 122–139 µ; adanal setae situated at level of posterior 1/3 of anal orifice; distance between adanal setae 49–54 µ; adanal setae setaceous and 33–40 µ long; distance from anterior margin to adanal setae ca. 64 µ; distance from adanal setae to postanal seta 37–55 µ; postanal seta very long, 124–137 µ. Shorter setae of unarmed venter 38–52 µ long with longer setae 56–66 µ long.

Stigma of peritreme (Fig. 22) located lateral to level between coxae III and IV with no peritremala extension posteriorly; anterior extension of peritreme narrowing somewhat and reaching to level of mid coxae I; total length of peritreme *ca.* 384 µ.

*Legs*: The length and width of leg segments is given in table 4. All measurements were made ventrally. Length measurements were made mid-ventrally and widths were made at the widest point. The measurements are approximate because there is about a 5–10% variation in measurements from specimen to specimen.

The leg chaetotaxy for deutonymphs is shown in table 5. The chaetotaxy formula for each leg segment is as follows:

al — d/v — pl (anterior laterals — dorsals/ventrals — posterior laterals). The total for each leg segment is in parenthesis bolded.

Leg I: Coxae I greatest width *ca.* 79 µ and mid–ventral length *ca.* 100 µ. Coxae I setae setaceous; proximal setae longer (*ca.* 54 µ) and distal setae shorter (*ca.* 49 µ). Proximal ventral seta of trochanter slender setaceous *ca.* 40 µ long and distal seta *ca.* 48 µ long. Distal dorsal setae of femur setaceous but with one short (*ca.* 20 µ long) and the other somewhat longer (*ca.* 32 µ). Proximal–most dorsal seta of genu *ca.* 30 µ long and central setae *ca.* 42 µ long. Tarsus with setae all setaceous.

Leg II: Coxae II greatest width *ca.* 135 µ and mid–ventral length *ca.* 45 µ. Anterior seta setaceous and *ca.* 39 µ long; posterior seta setaceous and longer, *ca.* 54 µ. Ventral setae of trochanter setaceous but with proximal seta much longer (*ca.* 50 µ) than distal (*ca.* 35 µ). Distal dorsal setae of femur stout, setaceous and short (*ca.* 43 long µ). Proximal anterior dorsal seta of genu short setaceous (*ca.* 30 µ long), whereas posterior seta longer setaceous (*ca.* 52 µ long). Tarsus with pre–apical seta all setaceous.

Leg III: Coxae III greatest width *ca.* 121 µ and mid–ventral length *ca.* 61 µ. Both coxal setae setaceous; anterior seta shorter (*ca.* 35 µ long) than posterior seta (*ca.* 59 µ long). Posterior proximal setae of trochanter setaceous and long (*ca.* 81 µ long); posterior distal seta shorter, slender setaceous (*ca.* 27 µ long). Setae of femur III stout, long setaceous (*ca.* 81 µ long). Proximal 2 setae of genu setaceous and subequal in length (51–60 µ). Tarsus with pre–apical seta setaceous.

Leg IV: Coxae IV greatest width *ca.* 101 µ and mid–ventral length *ca.* 61 µ. Single seta setaceous and 40–46 µ long. Dorsal proximal setae of trochanter setaceous (*ca.* 56 µ long) and distal setae shorter (*ca.* 38 µ) long. Dorsal distal setae of femur long setaceous (*ca.* 103 µ); setae of genu IV setaceous and subequal in length (43–48 µ); tarsus IV with pre–apical setae setaceous.

**Table 2.** Length/width (in µ) of leg segments for females.

**Segment**	**Leg I**	**Leg II**	**Leg III**	**Leg IV**
Trochanter	47/42	46/63	56/45	65/46
Femur	59/43	63/59	54/34	69/40
Genu	56/38	50/52	31/35	51/34
Tibia	60/35	38/45	32/41	47/35
Tarsus	100/30	85/31	98/26	228/39

**Table 3.** Leg segmental chaetotaxy for females.

**Segment**	**Leg I**	**Leg II**	**Leg III**	**Leg IV**
Trochanter	1 — 1/3 — 1 (**6**)	1 — 1/2 — 1 (**5**)	1 — 1/3 — 1 (**6**)	1 — 1/3 — 1 (**6**)
Femur	2 — 4/4 — 1 (**11**)	2 — 3/3 — 2 (**10**)	2 — 2/2 — 1 (**7**)	2 — 2/1 — 1 (**6**)
Genu	2 — 6/3 — 2 (**13**)	2 — 4/2 — 2 (**10**)	2 — 4/1 — 1 (**8**)	2 — 4/2 — 1 (**9**)
Tibia	2 — 6/3 — 2 (**13**)	1 — 4/3 — 2 (**10**)	2 — 4/1 — 1 (**8**)	2 — 4/3 — 1 (**10**)
Tarsus	3 — 6/6 — 3 (**18**)	3 — 4/4 — 3 (**14**)	3 — 4/5 — 3 (**15**)	3 — 4/5 — 3 (**15**)

**Table 4.** Length/width (in µ) of leg segments for Deutonymphs.

**Segment**	**Leg I**	**Leg II**	**Leg III**	**Leg IV**
Trochanter	67/58	67/87	70/90	103/94
Femur	117/61	108/98	92/72	147/67
Genu	111/57	54/88	56/68	92/63
Tibia	141/56	73/88	73/64	110/58
Tarsus	191/47	166/48	183/45	254/43

**Table 5.** Leg segmental Chaetotaxy for Deutonymphs.

**Segment**	**Leg I**	**Leg II**	**Leg III**	**Leg IV**
Trochanter	1 — 1/3 — 1 (**6**)	1 — 1/2 — 1 (**5**)	1 — 1/2 — 2 (**6**)	1 — 1/2 — 1 (**5**)
Femur	1 — 3/4 — 1 (**9**)	3 — 6/4 — 2 (**15**)	1 — 3/1 — 1 (**6**)	3 — 3/1 — 1 (**8**)
Genu	2 — 6/3 — 2 (**13**)	2 — 5/3 — 2 (**12**)	2 — 4/2 — 1 (**9**)	2 — 5/2 — 1 (**10**)
Tibia	2 — 6/4 — 2 (**14**)	1 — 4/3 — 2 (**10**)	2 — 3/2 — 1 (**8**)	2 — 4/2 — 2 (**10**)
Tarsus	4 — 8/5 — 4 (**21**)	3 — 5/4 — 3 (**15**)	3 — 5/5 — 3 (**16**)	3 — 3/5 — 3 (**14**)

## Discussion

*Haemogamasus alongipilis* was frequently collected along with another mite species: *Androlaelaps* (*Eubrachylaelaps*) *abrothrix* Gettinger and Lareschi (2009). From the 77 collections of *Haemogamasus alongipilis*, 37 collections (48%) also contained *Eubrachylaelaps abrothrix*. Three other mite species were found to occur less frequently with *Haemogamasus alongipilis*: An unidentified *Haemolaelaps* species, in 12 collections (15.6%); an *Ornithonyssus* species, in 9 collections (11.7%); and a Laelapidae species [#3], in 5 collections (6.5%). Four other mite species were occasionally found in collections with *Haemogamasus alongipilis*: A species of *Mysolaelaps* (2 collections); a species of *Gigantolaelaps* (1 collection); and two Laelapidae species, one with 2 collections and another with only 1 collection.

There were a total of 128 *Haemogamasus alongipilis* specimens mounted and identified. Of these, 33 (25.8%) were ♀s and 95 (74.2%) were deutonymphs. There were no male specimens found in the collections, likely because males are rare compared to females and deutonymphs. For the 3 *Abrothrix* host species there were 110 *Haemogamasus alongipilis* specimens mounted and identified. Of these 30 (27.3%) were ♀s and 80 (72.7%) were deutonymphs. Because *Haemogamasus* mites are primarily nest parasites and the deutonymphs are the migrating stage, the ratio (about 1:3) of females to deutonymphs in the collection is not surprising.

Table 6 below lists the *Haemogamasus alongipilis* collection records for each of the 7 host species collected in the 3 Argentina provinces. Most of the collections of *Haemogamasus alongipilis* were collected from *Abrothrix* rodents (84.4%), almost exclusively from *Abrothrix longipilis* (81.8%), with only 1 collection each from *Abrothrix olivaceus* and *Abrothrix xanthorhinus*. The 4 non–*Abrothrix* host species account for only 12 collections (15.6%). The 2 non–*Abrothrix* host species with 5 collections of *Haemogamasus alongipilis* each (*Geoxus valdivianus* & *Loxdontomys micropus*) are definitely valid records. The other 2 non–*Abrothrix* host species (*Oligoryzomys longicaudatus* & *Dromiciops gliroides),* with only 1 collection of *Haemogamasus alongipilis* each, are most likely incidental or accidental.

Table 7 lists the number and incident frequency of *Haemogamasus alongipilis* specimens collected from each of the 7 host species. For *Abrothrix longipilis* there were 135 host animals sampled with a total of 63 (46.7%) specimens of *Haemogamasus alongipilis* identified. Of the remaining 6 host species only 14 specimens of *Haemogamasus alongipilis* were identified. From the total of 224 host animals collected, 34.4% was found to host the 77 collections of *Haemogamasus alongipilis*.

Our study shows that the range of *Haemogamasus alongipilis* extends over more than three degrees of latitude (39°2' to 42°5'S) and a distance of 400 kilometers. All of the localities where the mice had this mite are located within the Valdivian rain forest phytogeographical region (Veblen et al. 1983). Presumably the mites occur on the same rodent species on the western side of the Andes Mountains in Chile in this same floristic region, thus is probably not of special conservation concern due to a small geographical distribution. But the total extent of the range of this mite remains to be determined. The main host species, *Abrothrix longipilis*, has a much larger total range than where we found this new mite. Further studies of the ectoparasites of this rodent will be required to determine whether the mite and this host have co–equal ranges. We have one limited bit of information suggesting that the mite may not co–occur with all populations of *Abrothrix longipilis*. Four individuals of this mouse that were collected at Parque Nacional Laguna Blanca (39°3'S, 70°21'W) did not have *Haemogamasus alongipilis*. This national park is located in the Occidental District of the Patagonian semi–desert region (Soriano 1983) that is characterized by the growth of perennial bunch grasses and low–growing shrubs, rather than trees. In this arid environment the *Abrothrix longipilis* are limited to microhabitats of dense growths of the bunch grasses or along the margins of isolated streams and seepages. The absence of *Haemogamasus alongipilis* from these four *Abrothrix longipilis* collections could be because the mite cannot survive the off–host phase of its life cycle in this general environment that is much less humid than that of the Valdivian rain forests.

A majority of the described species of *Haemogamasus* have been reported only from Europe and Asia. Of the twelve species reported from the western hemisphere, only 2 (*Haemogamasus ambulans* & *Haemogamasus pontiger*) are known to occur worldwide, and in North America from most of Canada and the United States. The other 10 species have been reported exclusively from North America.

The new species described herein, *Haemogamasus alongipilis,* is the first *Haemogamasus* species described from South America. However, there is one literature report of *Haemogamasus pontiger* (Berlese) having been collected in South Ameri*ca.* This report is from Argentina by Silvia Mosquera (1988) in the publication: Neotropica 34 (91): 73–76. The translated title of the paper is: “*Eucheyletia hardyi* Baker, 1949 and *Haemogamasus pontiger* (Berlese, 1904), two new mites for the Argentina fauna.” The citation of the *Haemogamasus* mite as given in this Spanish language paper is: “*Laelaps (Eulaelaps) pontiger* Berlesse, 1903 Redia 1: 260” followed by “*Haemogamasus pontiger* Baker et. al. 1956 Tech. Publ. Nat. Pests Control Assn.: 49”. This report contains a brief description with no illustrations. When Keegan’s (1951) description of the ♀ is compared with Mosquera’s Argentina ♀ description, this appears to be a valid but incidental record. Keegan reported the distribution of *Haemogamasus pontiger* to be cosmopolitan, and that it is apparently a facultative parasite, often free-living and associated with various animal nests. He also stated that *Haemogamasus pontiger* “were found in a variety of habitats: Wheat straw, flax tow, rice straw, rice hulls, in sod, and associated with the clothes moth.” Mosquera reported that the Argentina specimens were found free living in association with Acaridae mites feeding on grains and stored products. Thus, it is likely that this species does not occur as parasitic on small mammals in Argentina, but rather was introduced into the country with imported grains and stored products from elsewhere.

**Table 6.** Collection records by host and province.

**Province →Host species**	**Chubut**	**Neuquén**	**Río Negro**	**Total**
*Abrothrix longipilis*	17	16	30	**63**
*Abrothrix olivaceus olivaceus*	0	1	0	**1**
*Abrothrix olivaceus xanthorhinus*	0	1	0	**1**
*Geoxus valdivianus*	0	3	2	**5**
*Loxdontomys micropus*	2	1	2	**5**
*Oligoryzomys longicaudatus*	0	0	1	**1**
*Dromiciops gliroides*	0	0	1	**1**
**Total**	**19**	**22**	**36**	**77**

**Table 7.** Incident frequency of *Haemogamasus alongipilis* for each host species.

**Host species**	**Total Hosts Sampled**	***Haemogamasus alongipilis*Collected**	**FrequencyRate**
*Abrothrix longipilis*	135	63	46.7%
*Abrothrix olivaceus olivaceeus*	6	1	16.7%
*Abrothrix olivaceus xanthorhinus*	16	1	6.3%
*Geoxus valdivianus*	12	5	41.7%
*Loxdontomys micropus*	18	5	27.8%
*Oligoryzomys longicaudatus*	33	1	3.0%
*Dromiciops gliroides*	4	1	25.0%
**Total**	**224**	**77**	**34.4%**

## Supplementary Material

XML Treatment for Haemogamasus alongipilis
